# Current Findings from the Japan National Health and Nutrition Survey

**DOI:** 10.3390/nu15092213

**Published:** 2023-05-06

**Authors:** Nobuo Nishi, Hidemi Takimoto

**Affiliations:** 1 Graduate School of Public Health, St. Luke’s International University, Tokyo 104-0045, Japan; 2 National Institute of Health and Nutrition, National Institutes of Biomedical Innovation, Health and Nutrition, Osaka 566-0002, Japan; thidemi@nibiohn.go.jp

In this Special Issue, six articles using the Japan National Health and Nutrition Survey (NHNS) were published. Among the six articles, two examined the time trends [1,2], two used simulation models [3,4], one used cross-sectional data [5], and the other employed a qualitative method [6].

In Japan, the prevalence of hypertension has decreased with the improvement of medical treatment and decreased dietary salt intake. In panel data analysis, the trends in hypertension prevalence, treatment, and control were investigated by life expectancy at the prefectural level [1]. It was concluded that reducing the prevalence of hypertension through improving lifestyle factors, such as salt intake in each prefecture, may be important to decrease the disparity in life expectancy among prefectures.

Using the Joinpoint Regression Program, trends in food group intake were evaluated by physical size in young Japanese women in NHNS 2001–2019 data [2]. A decreasing trend in the intake of fish and shellfish and seaweed and an increasing trend in the intake of meat and soft drink were found in young women. Decreasing trends in the intake of fruit and dairy products were found in young women without obesity, while an increasing trend in the intake of confectionaries was found in young women with obesity. This study suggested that the patterns of unhealthy food intake may be different by physical size in young Japanese women.

In the super-aged society of Japan, it is expected that reducing dietary salt intake prevents cardiovascular disease and hence curbs growing healthcare expenditures. The effect of achieving global and national targets of salt reduction on cardiovascular events and national healthcare expenditures was estimated [3]. Compared with the status quo, reducing mean dietary salt intake towards the targets over 10 years would prevent 1–3% of events of ischemic heart disease and stroke and save up to 2% of related national healthcare expenditures. Achieving goals of salt reduction would yield modest health and economic benefits in Japan.

Japan experienced a decrease in cardiovascular mortality concurrently with a reduction in salt intake of the population since the 1950s. The impact of salt intake reduction on the long-term trends in cardiovascular mortality was estimated [4]. Compared to the base run, 298,000 and 118,000 excess deaths were found in men and women, respectively, on the assumption that dietary salt intake had not changed over the period. According to the model, the reduction in salt intake since the 1950s has contributed to a considerable decrease in cardiovascular mortality.

The association between sources of free sugars and weight status among children and adolescents was examined in a cross-sectional study using the 2016 NHNS [5]. It was unlikely that consuming free sugars from any food had an adverse effect on weight status among those who had a relatively low intake of free sugars.

In the NHNS, efforts are made to raise participation rates to ensure representativeness. Local government personnel in charge of NHNS were invited to discuss the measures to improve participation rates. The following measures were identified: standardization of survey methods, skills of survey staff, survey organization, venue setting, accessing target households, time of the survey, responses during the survey, confirming meal contents reported in the dietary intake survey, rewards/incentives, possible rewards, feedback on survey results, and practices during the COVID-19 pandemic [6]. These results show practicable initiatives for local health personnel to raise participation rates.

These findings will help readers understand the current situation of health and nutrition of the Japanese population.
